# Case Study: Harnessing Art Therapy for Patients With Learning Disability

**DOI:** 10.1192/bjo.2025.10708

**Published:** 2025-06-20

**Authors:** Sanskriti Babhulkar, Caroline Deodhar

**Affiliations:** Norfolk & Suffolk NHS Foundation Trust, Ipswich, United Kingdom

## Abstract

**Aims:** Individuals with learning disabilities (LD) have a higher rate of mental health disorders and behavioural difficulties.

Conventional interventions may be limited in addressing non-verbal emotional expression.

Art therapy offers a creative, structured medium for self-expression, emotional regulation and social skill development.

**Methods:** Case Report

A 42-year-old lady, Ms X, with moderate LD with challenging behaviour, paranoid schizophrenia and past history of mixed anxiety and depressive disorder and impact of art therapy in her treatment and quality of life.

Ms X has gone through a series of unfortunate events, for example, her brother committed suicide by jumping off a bridge. She lost her mother and her father had been diagnosed with Alzheimer’s dementia. Considering her level of LD and the trauma that she has gone through and the past history of risky behaviours including verbal and physical aggression, requiring inpatient admission and intensive support, she has been doing well.

There are times she tends to become increasingly anxious needing reassurance, however, these are more infrequent now. In general, she has been compliant with the prescribed medications, Depakote and olanzapine. She goes out for swimming quite frequently and also she enjoys walking along the beach with staff. She keeps herself active.

A significant improvement in her presentation is attributed to the art therapy she has been having regularly since September 2024–January 2025. She has completed 12 sessions and a remarkable positive change has been noted by staff supporting her.

**Results:** Discussion.

1. Improvement in Psychosocial Functioning.

A systematic review (37 studies) indicated that art therapy interventions significantly enhanced psychosocial well-being.

Key therapeutic factors included varied artistic mediums and therapist-guided sessions.

2. Reduction of Aggression.

Ms X had reduced episodes of aggression which is also supported by a quasi-experimental study (Egypt) that found 85% of participants exhibited reduced aggression.

3. Alleviation of Mental Health Symptoms.

Art therapy provides a non-verbal mode of expression, effective in treating patients with learning disabilities and comorbid anxiety/depression. Shown to improve emotional processing and engagement in therapy.

4. Enhancement of Social Skills.

Studies indicate that art therapy significantly improves social interactions, particularly in patients with learning disabilities.

**Conclusion:** Art therapy is a valuable adjunct that provides alternative communication channel, especially for non-verbal or emotionally withdrawn individuals.

Clinical Implications and Future Directions:

Incorporate art therapy into multidisciplinary-treatment plans for patients with learning disabilities.

Investigating neurobiological mechanisms underpinning art therapy’s impact could optimise therapeutic approaches.

